# Survival of >20 years in a myeloma patient with an unusual combination of t(14;16) and hyperdiploidy: A case report

**DOI:** 10.3892/ol.2013.1624

**Published:** 2013-10-11

**Authors:** CHOR SANG CHIM, EDMOND SIU KWAN MA

**Affiliations:** 1 University Department of Medicine, Queen Mary Hospital, University of Hong Kong, Hong Kong, SAR, P.R. China; 2 Department of Pathology, Hong Kong Sanatorium Hospital, Hong Kong, SAR, P.R. China

**Keywords:** t(14;16), hyperdiploidy, myeloma, survival

## Abstract

The current case report presents the prolonged survival of >20 years in a myeloma patient with secondary plasma cell leukemia and myelomatous pleural effusion. FISH on marrow plasma cells showed hyperdiploidy and concomitant t(14;16) and karyotypes predicting superior and short survival. The possibility of primary hyperdiploidy with t(14;16) as a secondary event has been discussed.

## Introduction

Myeloma remains an incurable disease despite a marked improvement of complete remission and survival rates. Median survival rates have improved from 2–3 to 7–8 years following the advent of novel agents (including thalidomide, lenalidomide and bortezomib) and autologous stem cell transplantation (ASCT) (1). Prolonged survival may occur in patients with low international staging system (ISS) stage of disease, good-risk karyotypic aberrations, including hyperdiploidy or t(11;14) or patients preceded by monoclonal gammopathy of unknown significance (2). Written informed consent was obtained from the patient.

## Case report

**Figure 1:**
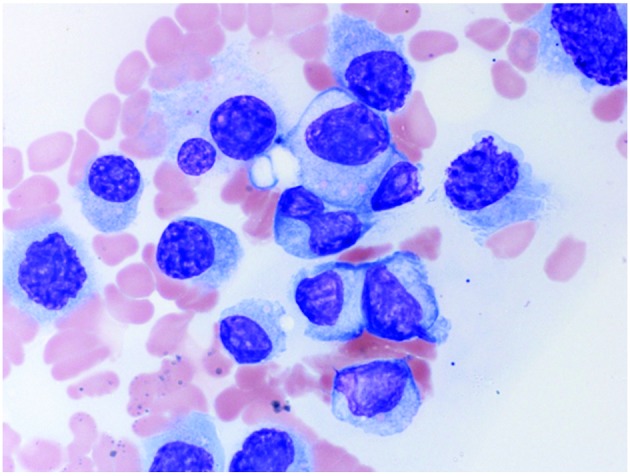
Bone marrow aspirate performed upon referral in 2012. Replacement of normal hematopoiesis by sheets of abnormal pleomorphic plasma cells that were large in size and featured round to lobulated nuclei outlines, open chromatin texture, distinct 1–3 nucleoli and abundant basophilic cytoplasm lacking a Golgi zone, was observed (Wright-Giemsa; magnification, ×1,000).

**Figure 2:**
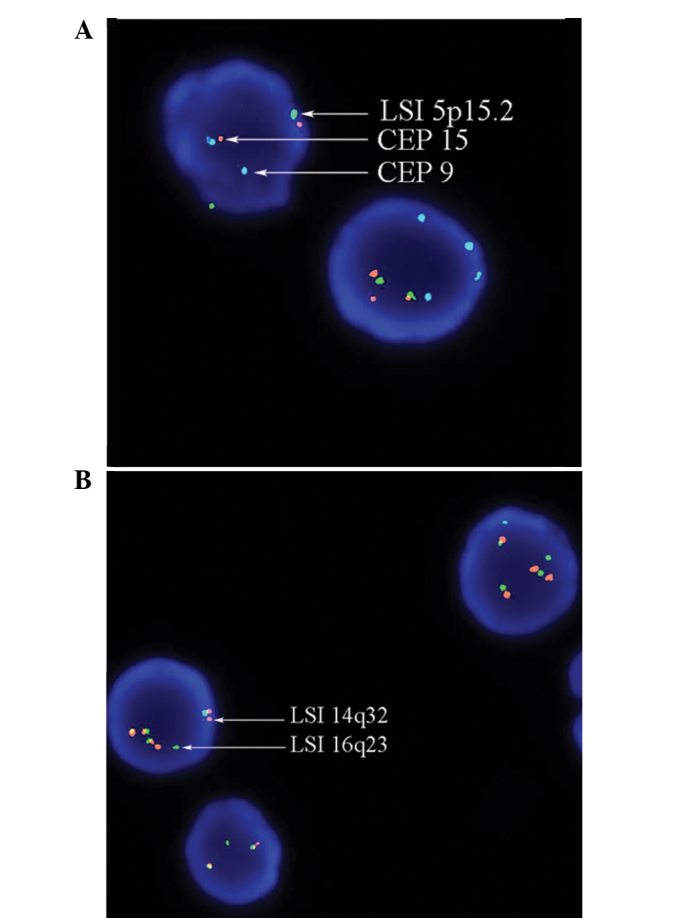
Interphase FISH. (A) Triploid pattern is shown in the myeloma cell on the right, i.e. three copies each of chromosomes 5, 9 and 15. (B) t(14;16) was found to be positive, as two yellow fusion signals against a background of polysomy were identified for chromosomes 14 and 16, i.e. 4 additional copies of chromosome 14 and 2 additional copies of chromosome 16. The presence of polysomy 14 was also confirmed by a t(4;14) probe (data not shown).

A 56-year-old male was referred for symptomatic myeloma in February 2012. The patient was first diagnosed with myeloma in April 1991, presenting with syncope and a hemoglobin (Hb) level of 6.5 g/dl. The patient exhibited partial response to melphalan-based regimens, including melphalan and prednisolone (MP), and vincristine, cyclophosphamide, melphalan and prednisolone (VCMP); serum IgG levels dropped from 8,750 mg/dl (normal, 819–1,725 mg/dl) at diagnosis to 2,020 mg/dl in February 1992. Chemotherapy was stopped in 1993 prior to the plateau phase with extremely good partial remission. Chemotherapy was resumed intermittently from 1996–1998. In 2002, VBCMP was used followed by MP maintenance. In late 2005, thalidomide was added to intermittent MP. The patient was referred to Queen Mary Hospital (Hong Kong, China) in December 2011. Upon referral, the patient’s serum albumin was 30 g/l and globulin was 76 g/l. Serum creatinine was 101 mg/ml (normal, 67–109 nmol/l) and calcium was 2.61 mg/dl (normal, <2.5 mg/dl). Serum IgG measured 6,150 mg/ml (normal, 819–1,725 mg/dl), IgA was 21 mg/dl (normal, 70–386 mg/dl) and IgM was 31 mg/dl (normal, 55–307 mg/dl). Serum immunofixation showed monoclonal IgG/λ with a paraprotein level of 52 g/l. Complete blood count showed a Hb count of 7.5 g/dl, platelet count of 30×10^9^/l (normal, 150–400×10^9^/l) and leukocyte count of 4.8×10^9^/l (normal, 4–11×10^9^/l) with blood smear showing 26% circulating plasma cells and, hence, secondary plasma cell leukemia. Bone marrow aspiration showed hypercellular bone marrow with 84% sheets of small- to large-sized CD138^+^ plasma cells and marked suppression of hematopoiesis (Fig. 1). Fluorescence *in situ* hybridization with a panel of probes for ploidy, t(4;14), t(14;16), del(17p) and chromosome 1q gain showed hyperdiploidy together with t(14;16) (Fig. 2). The patient was administered bortezomib, and cyclophosphamide and dexamethasone (VCD) was added. The patient developed left pleural effusion in July 2012, which showed pleomorphic plasma cells. Upon discussion with the family, VCD was switched to bortezomib, lenalidomide and dexamethasone (VRD). In late July 2012, the patient was admitted due to malaise and recurrent left pleural effusion, for which chemical pleurodesis was performed. The patient succumbed to refractory myeloma in December 2012.

## Discussion

In the present case report, although ISS and karyotyping were unavailable at diagnosis, a prolonged survival of >20 years without ASCT or bortezomib is rare. The presence of hyperdiploidy may explain the prolonged survival, even without ASCT or the use of novel agents, such as bortezomib or lenalidomide.

In addition, t(14;16) has been associated with poor prognosis and typically inferior survival (2). In the present patient, the diagnostic marrow was unavailable and, hence, it was not certain whether t(14;16) was present at diagnosis or acquired later on, during the course of disease. In a previous study of additional cytogenetic aberrations in hyperdiploid myeloma at diagnosis (3), it was shown that hyperdiploidy with concomitant t(14;16) occurs rarely at diagnosis, which has been associated with poor survival (3). Therefore, if t(14;16) occurred at diagnosis, the present patient may have exhibited considerably shorter survival. Thus, t(14;16) may have been acquired during the course of the patient’s myeloma, which has not been previously reported.

The current patient presented at follow-up in Feb 2012. The value of circulating plasma cells in the peripheral blood was 26%, consistent with secondary plasma cell leukemia. The extramedullary disease nature was further illustrated by the refractory malignant pleural effusion prior to the patient’s demise. Secondary genetic aberrations, such as TP53 deletion and/or 1q23 amplification, may develop at chemoresistant relapse or terminal myeloma with extramedullary disease (4,5). However, in the current study, del(17p) and amp(1q) were absent and, hence, the genetic events triggering the extramedullary myeloma remain unknown.

In summary, the current case report presents the prolonged survival of >20 years in a myeloma patient with secondary plasma cell leukemia and myelomatous pleural effusion. FISH on marrow plasma cells showed hyperdiploidy and concomitant t(14;16), and karyotypes predicting superior and short survival. The possibility of a primary hyperdiploidy with t(14;16) as a secondary event has been discussed. While hyperdiploidy in myeloma usually predicts superior survival, additional IgH translocation may adversely impact prognosis. The possibility of t(14;16) as a secondary genetic event requires further investigation.
